# Saudi Arabian Plants: A Powerful Weapon against a Plethora of Diseases

**DOI:** 10.3390/plants11243436

**Published:** 2022-12-08

**Authors:** Hesham R. El-Seedi, Safaa M. Kotb, Syed G. Musharraf, Awad A. Shehata, Zhiming Guo, Sultan M. Alsharif, Aamer Saeed, Omer A. A. Hamdi, Haroon Elrasheid Tahir, Rasha Alnefaie, Rob Verpoorte, Shaden A. M. Khalifa

**Affiliations:** 1 Pharmacognosy Group, Department of Pharmaceutical Biosciences, Biomedical Centre, Uppsala University, P.O. Box 591, SE 751 24 Uppsala, Sweden; 2 International Research Center for Food Nutrition and Safety, Jiangsu University, Zhenjiang 212013, China; 3 International Joint Research Laboratory of Intelligent Agriculture and Agri-Products Processing, Jiangsu Education Department, Jiangsu University, Zhenjiang 212013, China; 4 Department of Chemistry, Faculty of Science, Menoufia University, Shebin El-Kom 32512, Egypt; 5 Department of Chemistry & Microbiology, Faculty of Science, Menoufia University, Shebin El-Kom 32512, Egypt; 6 H.E.J. Research Institute of Chemistry, International Center for Chemical and Biological Sciences, University of Karachi, Karachi 75270, Pakistan; 7 Avian and Rabbit Diseases Department, Faculty of Veterinary Medicine, University of Sadat City, Sadat City 32897, Egypt; 8 School of Food and Biological Engineering, Jiangsu University, Zhenjiang 212013, China; 9 Biology Department, Faculty of Science, Taibah University, Al Madinah 887, Saudi Arabia; 10 Department of Chemistry, Quaid-I-Azam University, Islamabad 45320, Pakistan; 11 Department of Chemistry, Faculty of Science, University of Khartoum, Khartoum 11115, Sudan; 12 Department of Biology, Faculity of Science, Al-Baha University, Albaha 65779, Saudi Arabia; 13 Natural Products Laboratory, Institute of Biology, Leiden University, P.O. Box 9505, 2300RA Leiden, The Netherlands; 14 Department of Molecular Biosciences, The Wenner-Gren Institute, Stockholm University, SE 106 91 Stockholm, Sweden

**Keywords:** Saudi Arabian plants, distribution, anti-inflammation, anti-cancer, antibiotic resistance, active compounds

## Abstract

The kingdom of Saudi Arabia (SA) ranks fifth in Asia in terms of area. It features broad biodiversity, including interesting flora, and was the historical origin of Islam. It is endowed with a large variety of plants, including many herbs, shrubs, and trees. Many of these plants have a long history of use in traditional medicine. The aim of this review is to evaluate the present knowledge on the plants growing in SA regarding their pharmacological and biological activities and the identification of their bioactive compounds to determine which plants could be of interest for further studies. A systematic summary of the plants’ history, distribution, various pharmacological activities, bioactive compounds, and clinical trials are presented in this paper to facilitate future exploration of their therapeutic potential. The literature was obtained from several scientific search engines, including Sci-Finder, PubMed, Web of Science, Google Scholar, Scopus, MDPI, Wiley publications, and Springer Link. Plant names and their synonyms were validated by ‘The Plant List’ on 1 October 2021. SA is home to approximately 2247 plant species, including native and introduced plants that belong to 142 families and 837 genera. It shares the flora of three continents, with many unique features due to its extreme climate and geographical and geological conditions. As plants remain the leading supplier of new therapeutic agents to treat various ailments, Saudi Arabian plants may play a significant role in the fight against cancer, inflammation, and antibiotic-resistant bacteria. To date, 102 active compounds have been identified in plants from different sites in SA. Plants from the western and southwestern regions have been evaluated for various biological activities, including antioxidant, anti-cancer, antimicrobial, antimalarial, anti-inflammatory, anti-glycation, and cytotoxic activities. The aerial parts of the plants, especially the leaves, have yielded most of the bioactive compounds. Most bioactivity tests involve in vitro assessments for the inhibition of the growth of tumour cell lines, and several compounds with in vitro antitumour activity have been reported. More in-depth studies to evaluate the mode of action of the compounds are necessary to pave the way for clinical trials. Ecological and taxonomical studies are needed to evaluate the flora of SA, and a plan for the conservation of wild plants should be implemented, including the management of the protection of endemic plants.

## 1. Introduction

The plants of Saudi Arabia (SA) have high biological diversity in the Arabian Peninsula, where they represent an important genetic resource for crop and medicinal plants. About 30% of these plants are rare due to the geographical location and dry weather characteristics of SA [1,2]. Various parts of the plants are used in traditional medicine, including the bark, flowers, fruits, leaves, resins, rhizomes, roots, seeds, and stems. All plants contain primary metabolites, while secondary metabolites serve a specific plant species in the interaction with its environment. The contents of biologically active compounds are plant-specific and genetically determined; however, they are also affected by the cultivation, practices, pests and diseases, climate, weather, stage of development (season, young and old leaves), ecosystem, and the time of the day the material is collected [3]. Because of the extreme conditions in SA, plants have developed different strategies to survive. In terms of phytochemistry, this results in high levels of secondary metabolites, such as flavonoids, polyphenols, terpenes, tannins, saponins, sterols, alkaloids, and their glycosides [3].

The importance of natural products for novel drug development is obvious from present-day pharmacology, and many natural products have served as the basis for developing medicines and are still used as medicines for various ailments. Morphine and codeine from *Papaver somniferum* L., quinidine, and quinine from *Cinchona* spp., atropine from *Atropa belladonna* L., and digoxin from *Digitalis* spp. are prominent examples of well-established plant-derived pharmaceutical preparations [4]. Newman and Cragg [5] demonstrated that almost one-half of all novel medicines developed in recent decades are natural products or natural product derivatives and analogues. Therefore, searching nature for novel hits for the development of medicine is increasing [6].

Novel medicines are required because of several problems with present-day pharmaceuticals, including severe side effects and resistance to anti-cancer medicines or antibiotics. For example, the common non-steroidal anti-inflammatory drugs (NSAIDs) are known for their side effects, such as cardiovascular incidents and gastrointestinal bleeding [7]. A recent study conducted in SA reported that the side effects of NSAIDs and antibiotics were reported by 80.7% (*n* = 272) and 48.7% (*n* = 164) of users, respectively [8]. Novel NSAIDs with fewer side effects are needed. In addition to the side effects of antibiotics, their uncontrolled use increases the likelihood of microorganisms developing resistance, and thus, increases the number of severe infections and mortality [9]. Cancer incidence in SA has increased in the past 27 years; thyroid cancer incidence increased by 26-fold and breast, bladder, colon, and uterine cancer incidences increased by about 10-fold. The number of cancer deaths in SA increased from 5% in 1990 to 12% in 2016 [10].

Saudi citizens are dependent on both traditional medicinal plants and modern medicines, as was reported in a review on the ethnopharmacology of SA plants [11]. Although several publications report on traditional medicines in SA [12,13,14], there is no comprehensive review about the total plant biodiversity in SA and its uses to date. Combining traditional knowledge with modern pharmacognostic research, novel hits for drug development may be found, resulting in the evidence-based use of traditional medicines. Other recent studies have shown interesting non-medical applications of secondary metabolites from Saudi Arabian plants. An example of a plant from SA with a high content of valuable compound(s) is *Plectranthus aegyptiacus*, a source of thymol, which is the main component of the plant’s essential oil (58.49%). Thymol is a volatile phenolic monoterpenoid; among other uses, it is used in food conservation [15,16,17,18]

The aim of this review is to evaluate the present knowledge on the plants growing in SA regarding their pharmacological and biological activities and the identification of bioactive compounds. A critical analysis of the published evidence should lead to the identification of plant species that are of interest for further studies of biologically active compounds. This could lead to the evidence-based application of traditional medicines and novel leads for drug development. Furthermore, other non-medical applications of SA plants and their constituents may result from the present knowledge. Therefore, this review should serve as the basis for selecting plants for further in-depth reviews of the active compounds previously reported in plants growing outside SA or plant compounds that have been previously isolated from other sources. A major effort to study biodiversity in SA was recently made by Alqethami et al. [3]. In their phytochemical screening of 85 different medicinal plant species used in Jeddah city for traditional medicine, glycosides (82%; 70 species) were the most common followed by tannins (68%; 58 species), alkaloids (56%; 48 species), saponins (52%, 44 species), and flavonoids (35%; 30 species) [3].

As part of our ongoing project on chemical and pharmacological studies of plants used in traditional medicine [19,20], in this review, we will focus on the NSAIDs, antibiotics, and antitumour compounds that have been reported in plants growing in SA. Cardiovascular disease, diabetes, and obesity targets deserve more attention.

## 2. Distribution and Diversity of the Flora of Saudi Arabia

SA is the largest country in the Arabian Peninsula, occupying about 2.25 million km^2^, or nearly 80% of the peninsula [21]. Forests cover about 27,000 km^2^ of its total area, mostly in the southwestern part of the Kingdom [22], while the area of natural rangeland exceeds 1.75 million km^2^ [23]. Plant diversity plays a key role in maintaining and preserving ecological balance and stability, which is an important factor for human well-being, not only for the country but for the whole world [24]. In recent years, a significant amount of data have been published about the flora in SA, which revealed that the country is gifted with a wide variety of plants, including a large number of herbs, shrubs, and trees that are found in desert, semi-desert, and mountainous ecosystems [25,26,27]. Geographic and climatic diversity, changes in water resources, drought, and anthropogenic pressures, such as overgrazing, deforestation, and all developmental activities in SA, negatively affect biodiversity, impacting the structure of the flora and natural vegetation [22,28]. The climate of SA varies greatly depending on the area and the season; it is characterised by extremes in weather, with very high temperatures during the daytime followed by an abrupt drop in temperature at night and very little, irregular precipitation (i.e., a typical ‘desert climate’) [21]. The annual mean temperature of the country is 24.64 °C, with Turaif in the north recording the lowest temperature (19.10 °C) and Makkah in the west recording the highest temperature (31.69 °C), with an average of 17.62 °C, exhibiting an increasing gradient from north to south [29]. Rainfall and temperature are considered the key controllers of agricultural production [30]. The annual average rainfall in SA from 1979 to 2009 was 93.5 mm, and it is expected that by 2050, some parts of SA will experience a decrease in rainfall by 20–25% in dry seasons and 10–15% in the winter. The maximum temperature has increased significantly, especially in dry seasons, by 0.72 °C per decade [31].

SA is home to about 2247 plant species, including native and introduced plants that belong to 142 families and 837 genera, and the components of its flora are the admixture of the features of Africa, Asia, and the Mediterranean region in addition to a large number of about 246 endemic species [32,33,34,35,36]. Of them, about 105 species reside in dunes, 90 species are halophytes, 75 species are trees, and aquatic plants comprise 12 species. About 450 species of total flora have a direct benefit to humans; 334 species have medicinal value, 38 species are palatable fodder, 25 species are human food, 45 species are poisonous, 47 species are used as ornamentals, and 471 species are used in ethnomedicine [22,37]. Of note, about 600 species of plants in SA are rare and endangered in their habitat [38].

**Figure 1:**
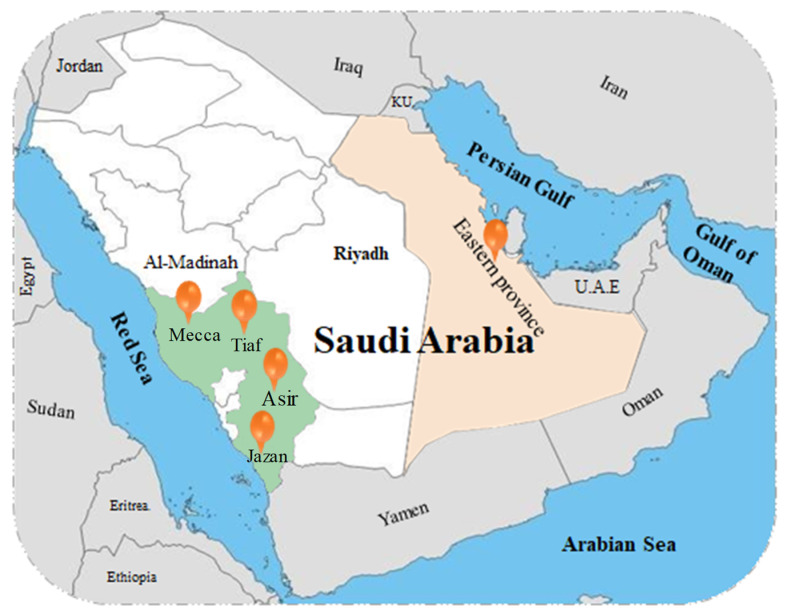
A map showing the surveys sites of different ecological areas in Saudi Arabia (SA).

Mecca city district is characterised by various types of plants; it was reported that a total number of 184 species of 125 genera and 44 families are found in Mecca. The most well-known families are Poaceae (17%), Fabaceae (13%), and Amaranthaceae (5%) [32]. Moreover, wadis represent one of the most notable desert landforms where physiographic irregularities appear, leading to parallel variation in species distribution and life forms. In wadi Al-Noman in Mecca, about 126 species from 39 families have been recorded, namely Fabaceae, Poaceae, and Boraginaceae. The authors indicated that species diversity in the studied area may be positively associated with the high pH and mineral content of the soil [39]. The vegetation along a transect between Makkah Al-Mukarramah and Al-Madinah Al-Munawarah in the Hejaz Mountains includes 106 species of vascular plants from 35 families [40]. In a survey of a sector in the Hejaz mountains along the Medina-Badr road, 247 species of 173 genera and 52 families are listed [41]. In Jizan province in the southern region, with varied landscapes such as wadis, islands, plateaus, and mountains, 850 species of 434 genera from 98 families represent about 37% of all the species in SA. Of them, 536 species are from Jabal Fayfa and 202 species are from the Farasan Islands. The most dominant families are Poaceae, Papilionaceae, and Asteraceae, with a total of 87, 70, and 60 species, respectively [42]. In a survey of the flora of the Sarwat Mountains in Taif as showed in Figure 1, 261 species from 178 genera and 55 families were recorded, of which Asteraceae, Poaceae, Fabaceae, Lamiaceae, Chenopodiaceae, Boraginaceae, Asclepiadaceae, Brassicaceae, and Zygophyllaceae were the most common [43]. In another mountainous area, the Asir mountains in the southwest of SA, 189 species of 74 families were recorded; Asteraceae was the dominating family in the study area (36.5%) followed by hemicryptophytes (15%) and geophytes (12.5%) [28]. In the eastern part of the Kingdom, no more than 370 native species of herbs and shrubs are adapted to the desert climate, reflecting the difficulties faced by plants in these harsh environments; the same applies for the northern part [44]. This is in contrast with Asir Hijaz, the western mountainous area of the Kingdom, which has large species diversity due to the greater rainfall [33]. The number of recorded species is still increasing through new biodiversity surveys [32]; however, the richness and diversity of the flora might be lost due to anthropogenic pressure and changes in climate [37,45]. Therefore, further studies are needed to ecologically and taxonomically evaluate all the flora of the country and to provide suitable conservation measures for these valuable species with unique features.

## 3. History of Medicinal Plants in Saudi Arabia

People have used plants to meet their various needs throughout different eras. Plants have formed the basis of traditional medicine for at least 6000 years in Mesopotamia and Egypt, with Hippocrates serving as a landmark for the written traditions on medicines [46,47]. Traditional medicine has been and is still a part of the culture of SA [48]. SA has some unique features besides its religious position. Zamzam Well is one of the most well-known places in SA, known for its suitable environment for many herbs and shrubs, as mentioned in the verses of the Qur’ân*: Our lord, I have settled some of my offspring in a barren valley, near your sacred House, our Lord, that they may establish prayer. So make the hearts of people incline towards them and provide them with sustenance that they may give thanks* **(Verse # 37, Surah Ibrahim, p. 260).**

Agriculture has been the main source of economic income for several Saudi regions, even during the pre-Islamic times, and farmers followed both advanced and traditional irrigation methods by diverting rainwater flowing into agricultural terraces or bringing rainwater drawn from dams to irrigate parched wadis [43]. From the 7th century, SA has been the historical origin of Islam. ‘Traditional Islamic medicine’ is the term used to describe medical traditions thriving during Islam’s golden age [49]. In fact, many plants are highly recommended by Prophet Muhammad (peace be upon him (PBUH)). Other plants are mentioned in different surahs of the Holy Qur’ân, such as date palm, olive, toothbrush tree, camphor tree, squash, ginger, onion, grapes, clover [50]. Prophet Muhammad (PBUH) mentioned the use of Miswak (*Salvadora persica* L.) for oral hygiene [51]. The Prophet (PBUH) said, *‘Siwak (Miswak) is a means of purification for the mouth and is pleasing to the Lord’* [52]. At that time, the use of medicinal plants to prevent and treat health disorders flourished, and philosophers and physicians, such as Al-Razi and Ibn Sina, began to change the concept of medicine. They compiled medical books from the knowledge available in the Mediterranean region (e.g., from Egyptian, Persian, Greek, and Roman times) that have been used for centuries (700–1500 AD). This knowledge was selected through controlled experimentation and sound reasoning. ‘The Canon of Medicine’, ‘The Book of Healing’, and ‘Kitab Al-Hawi’ are the most famous books written by Ibn Sina (Father of Early Modern Medicine) and Al-Razi [50,53].

For these reasons, people in SA have taken an interest in plants; about 80% of Saudi citizens have used medicinal plants for their primary health care [11]. Recent publications reported the activity of *S. persica* (Miswak, toothbrush tree) against oral bacteria that cause caries. This species showed effective antimicrobial, antiulcer, analgesic, anti-inflammatory, and antitumour activities, which agrees with the recommendations of the Prophet (PBUH) [54]. The uses of 12 medicinal plants that were recommended by the Prophet (PBUH) and mentioned in Qur’ânic verses were recently published [50,55].

## 4. Anti-Inflammatory Activity of Saudi Arabian Plants

People have always been exposed to inflammatory diseases; the clinical symptoms were first described by the Roman physician Cornelius Celsus in the first century A.D. [56]. Inflammation is an immune response to various external factors, such as infection with microorganisms, toxic compounds, damaged cells, or irradiation [57]. An inflammatory response is a multi-step process initiated by inducers that are detected by specific receptors (sensors) on specialised sentinel cells (e.g., mast cells, macrophages, and dendritic cells). Sensors stimulate the production of mediators, such as cytokines, chemokines, eicosanoids, bioactive amines, and products of proteolytic cascades, which affect target tissues [56]. Such inflammatory inducers may lead to acute and/or chronic inflammatory responses in various body organs (e.g., liver, heart, brain, lung, intestinal tract, kidney, pancreas, and reproductive system), resulting in tissue damage or disease [58,59]. Worldwide, three out of five people die due to chronic inflammation caused by cardiovascular diseases, cancer, stroke, bowel diseases, chronic respiratory diseases, arthritis, obesity, and diabetes [60]. Unfortunately, the available anti-inflammatory drugs are associated with a number of side effects, including cardiovascular effects and gastrointestinal bleeding [7]. Therefore, there is a need for new potent drugs with fewer side effects for better management of inflammatory ailments. Natural products, especially medicinal plants, are a rich source of phytochemicals, which have been shown to relieve pain or have anti-inflammatory effects [61,62,63]. For example, curcumin isolated from *Curcuma longa* L. was the subject of several clinical studies of inflammatory diseases [64]. In-depth studies are needed to identify the mode of action and to optimise the pharmacological profile of plant extracts and active compounds.

**Figure 2:**
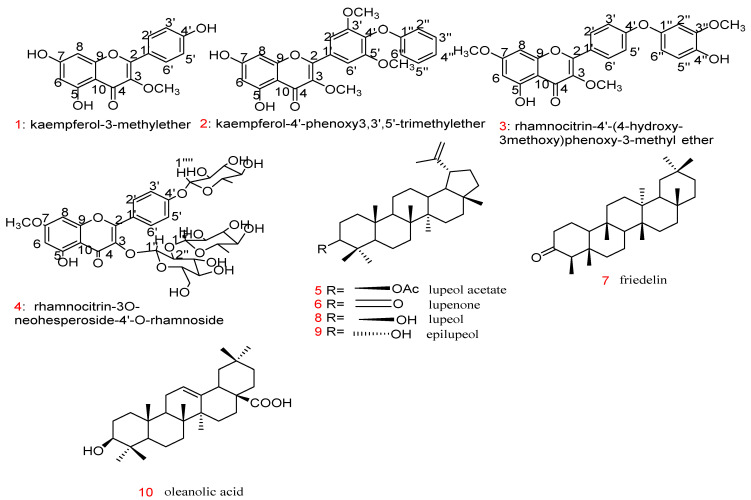
Structure of compounds 1 to 10 with anti-inflammatory effects.

Regarding the native plants of SA, several have been found to inhibit either the production of pro-inflammatory mediators or decrease their action, leading to anti-inflammatory activity [65]. Essential oils from *Achillea fragrantissima* Sch. Bip. and *Lactuca serriola* L., two plants of the family Asteraceae that grow in SA, were investigated for their anti-inflammatory activity; they were shown to exhibit a high inhibition of carrageenan-induced oedema after 4 h at a dose of 100 mg/kg orally by 71.9% and 73.4%, respectively [66]. The promising effects were due to the presence of azulene and oxazolidine in both *A. fragrantissima* and *L. serriola* [66]. Elsharkawy et al. [67] examined the anti-inflammatory effects of a mixture of three different plants (*Artemisia herba alba*, *Rubia tinctorum*, and *Alkanna tinctoria*) used by Bedouins in SA to treat many skin diseases. The oil mixture of these plants showed anti-inflammatory activity with oedema inhibition after 4 h (78.0% with 100 mg/kg orally) compared to the standard drug (89.5% with 10 mg/kg indomethacin) [67]. Using a bioassay-guided approach to identify the anti-inflammatory activity of compounds in *Cadaba glandulosa* Forssk, four active compounds were isolated and identified. The paw oedema thickness of rats was found to be 3.04, 3.11, 4.16, and 4.89 mm 4 h after the application of compounds **3**, **2**, **1**, and **4**, respectively, at 10 mg/kg subcutaneously compared with the standard drug (indomethacin 10 mg/kg reduced oedema thickness to 4.47 mm) [68]. Regarding the structure–activity relationship (SAR), compound **3** exhibited the highest anti-inflammatory activity in this study. The total MeOH extract (100 mg/kg) of *C. glandulosa* Forssk reduced the oedema thickness to 4.29 mm after 4 h [68]. Six triterpenes (compounds **5**–**10**) of *Kleinia odora* were evaluated for their anti-inflammatory activity, causing a decrease in pro-inflammatory genes like nuclear transcription factor-kB (NF-κB) and cascaded interleukins (IL-6, IL-1β, and TNF-α) [69]. Only compounds **5** and **6** produced a notable change, decreasing oedema thickness at a low dose of 0.4 μg/mL, but other compounds (**7–10**) showed only good anti-inflammatory activity at 40 μg/mL. Regarding skin SAR, the activity may be due to the lupane nucleus with ketone or acetate at position C-3, as in compounds **6** and **5** in Figure 2, respectively; however, the presence of an OH^-^ group at position 3 causes a decrease in activity, as for compounds **8** and **9** [69]. Hexane and chloroform extracts of *Huernia* Sp. Nov. aff. Boleana exhibited good activity, as the rats’ tail volume decreased and wound repair and re-epithelisation were enhanced [70]. Additional plants and active compounds with potent anti-inflammatory activity from the central and western regions of SA have been reported (Table 1).

## 5. Anti-Cancer Activity of Saudi Arabian Plants

**Figure 3:**
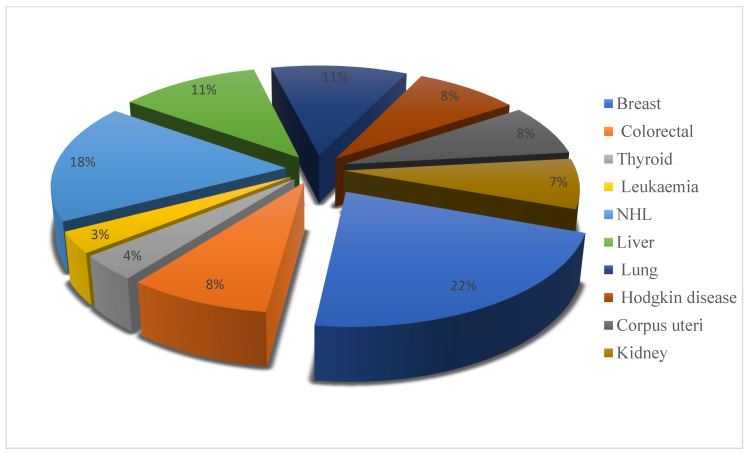
The 10 most common cancers in Saudi Arabia in 2012.

Cancer is an increasing problem, with a high incidence and high rate of mortality. Cancer is a general term for a number of chronic diseases commonly associated with the presence of malignant cells. Cells divide abnormally, with a lack of differentiation, and are unable to respond to normal physiological stimuli, quickly spread to the surrounding tissues [78,79,80]. Cancer can be caused by internal factors, including gene mutations, hormones, immune disorders, or other cellular changes, and external factors, such as tobacco, diet, infectious diseases, radiation, chemicals, or obesity [81]. According to global statistics, there were about 19.3 million new cancer cases and nearly 10 million deaths from cancer worldwide in 2020 [82,83]. The Saudi Cancer Registry estimated the total number of incident cases of cancer in 2012 to be 14,336 cancer cases, with 6791 among males and 7545 among females. Incidence varied by region, with the East and Riyadh showing the highest age-standardised rates for both males and females, and the lowest was in Hail and Jazan [84]. Breast (14.2%) and colorectal (11.1%) cancers were the most widespread cancers diagnosed in SA in 2012. The projection model predicted a fourfold increase in the incidence of colorectal cancer in SA in both sexes by 2030 [85]. The 10 most common cancers among Saudis are presented in Figure 3 [84]. 

Numerous conventional and advanced methods are used to treat and aid in cancer control, including radiation therapy, surgery, and chemotherapy. Nowadays, there are novel strategies for cancer control and treatment, including gene therapy, immunotherapy, photodynamic therapy, hyperthermia treatment, and hormone therapy [86,87]. Over 50% of the available anti-cancer drugs are natural products, modified natural products, or synthesised analogues [88,89,90]. Despite these advanced technologies and the extensive research that has been published recently, cancer remains a worldwide killer [91]. Anti-cancer drugs not only destroy cancer cells but also affect normal cells [92]; therefore, the search for natural products with no or lower side effects is ongoing.

Because plants have wide chemical diversity, their compounds provide a valuable opportunity to discover new drugs [6]. According to Newman and Cragg (2020), around two-thirds of anti-cancer drugs are derived from naturally occurring secondary metabolites [5]. For example, paclitaxel is a vital anti-cancer alkaloid that was isolated for the first time from the bark of the western yew (*Taxus brevifolia* Nutt.) and was characterised as part of the National Cancer Institute screening programme at the Research Triangle Institute [93]. 

**Figure 4:**
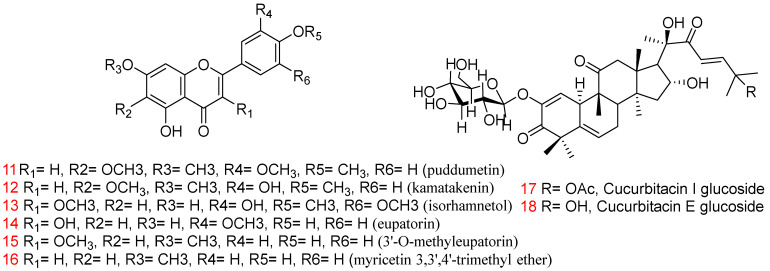
Structures of compounds **11–18** with anti-cancer activity.

The Kingdom of SA is rich in plants that have been reported to have anti-cancer and antioxidant activity as in Table 2 [18,37,94]. About 91 plant species from SA belonging to 38 families have exhibited anti-cancer activities against different human cancer cell lines. The plant families that have received the most attention due to their anti-cancer impact are Asteraceae (25%), Apocynaceae (10%), Fabaceae (7%), Boraginaceae (3%), and Brassicaceae (3%) [95]. For example, *Catharanthus roseus* L., which belongs to the Apocynaceae family, grows throughout in the Kingdom of SA, especially in the Makkah region, and it is known to contain more than 150 valuable alkaloids [96]. Vinblastine and vincristine; two important vinca alkaloids of *C. roseus*, are used as anti-cancer drugs [97]. The two compounds are used to treat human neoplasms; vinblastine is used to treat Hodgkin’s disease, lymphosarcoma, choriocarcinoma, neuroblastoma, carcinoma of breast, lungs, and other organs, and in acute and chronic leukaemia, while vincristine sulphate (Oncovin) arrests mitosis in metaphase in acute leukaemia in children, including lymphocytic leukaemia, Hodgkin’s disease, Wilkins’s tumour, neuroblastoma, and reticulum cell sarcoma [98]. In another study in which eight sesquiterpene lactones and six methoxylated flavonoids were isolated from *Artemisia sieberi* Besser (Asteraceae family), a plant growing in SA, the isolated flavonoids exhibited cytotoxic activity against human MCF-7 and HeLa cell lines. Compounds **11, 12**, and **13** as in Figure 4 exhibited the highest cytotoxic activity, with IC_50_ = 2.3, 3.9, 4.3 μg/mL, respectively, against HeLa cells and IC_50_ = 4.1, 3.2, 6.9 μg/mL, respectively, against MCF-7 cells (doxorubicin as a control at 0.40 and 0.69 μg/mL) compared with other flavonoids (14–16) [99]. In vitro and in vivo investigations of two compounds isolated from the fruits of *Citrullus colocynthis* L. Schrad. collected from the desert of Makkah, namely cucurbitacin I glucoside (**17**) and cucurbitacin E glucoside (**18**), showed strong cytotoxic activity against HepG2 cells with IC_50_ of 1.9 and 2.5 μg/mL, respectively. In addition to these activities, both compounds can prolong life span and survival time and regulate the biochemical parameters in vivo of rats that have Ehrlich’s ascites carcinoma tumours [100]. The methanol extracts of different parts of 11 plants from SA exhibited anti-cancer potential in a human liver cancer cell line, and the most active extracts of *Artemisia monosperma*, *Calligonum comosum*, *Ochradenus baccatus*, and *Pulicaria glutinosa* Jaub. and Spach were analysed to detect the presence of 41 phytomolecules, including the compounds 1-(2-hydroxyphenyl) ethenone, 3-(4-hydroxyphenyl) propionitrile, 8,11-octadecadiynoic acid methyl ester, and 6,7-dimethoxycoumarin, respectively [101]. These plant families may have unique anti-cancer properties. Therefore, other compounds occurring in these plants need in-depth studies to be applied as anti-cancer drugs.

## 6. Antibacterial Activity of Saudi Arabian Plants and Their Potential Contribution against Bacterial Antibiotic Resistance

**Figure 5:**
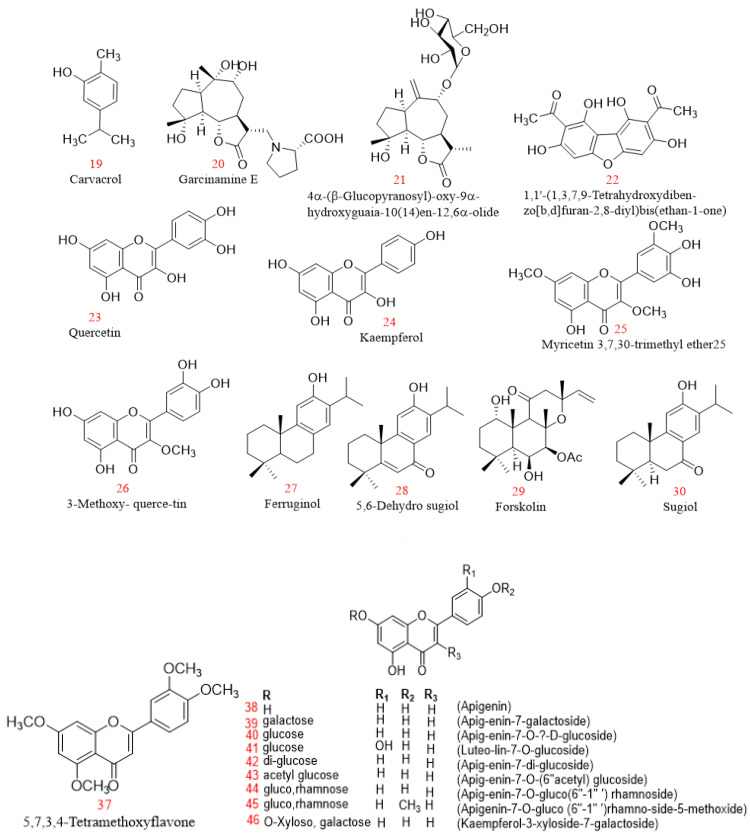

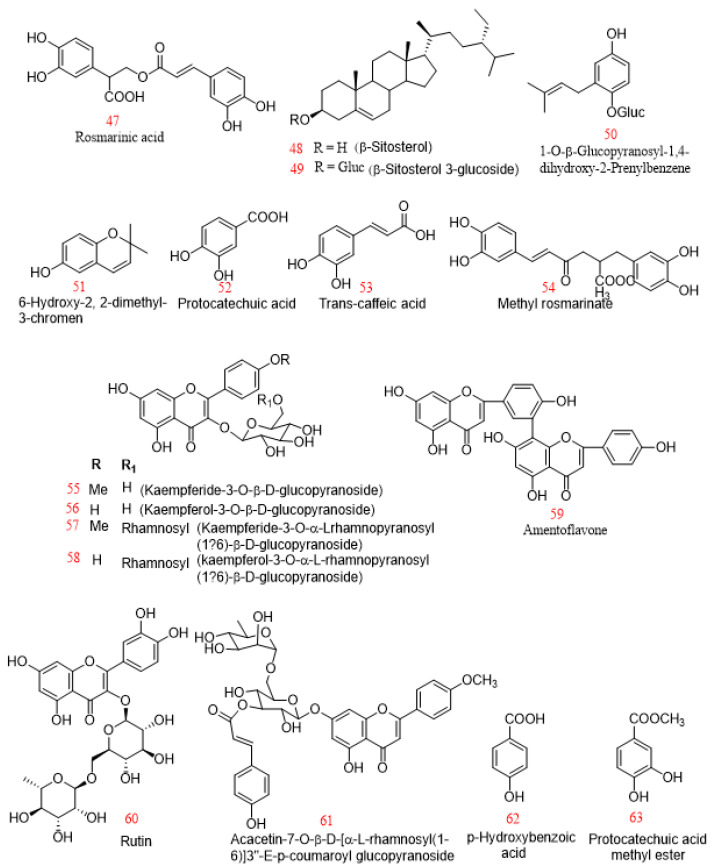

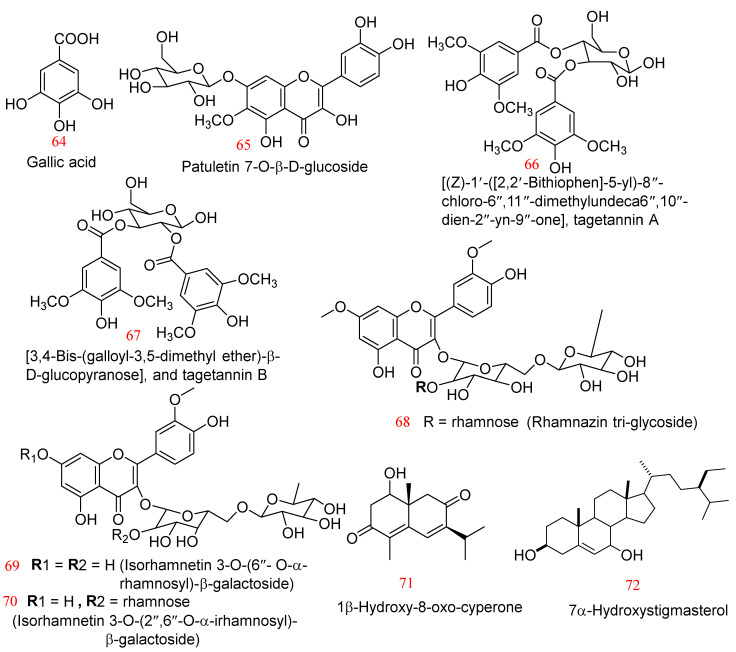

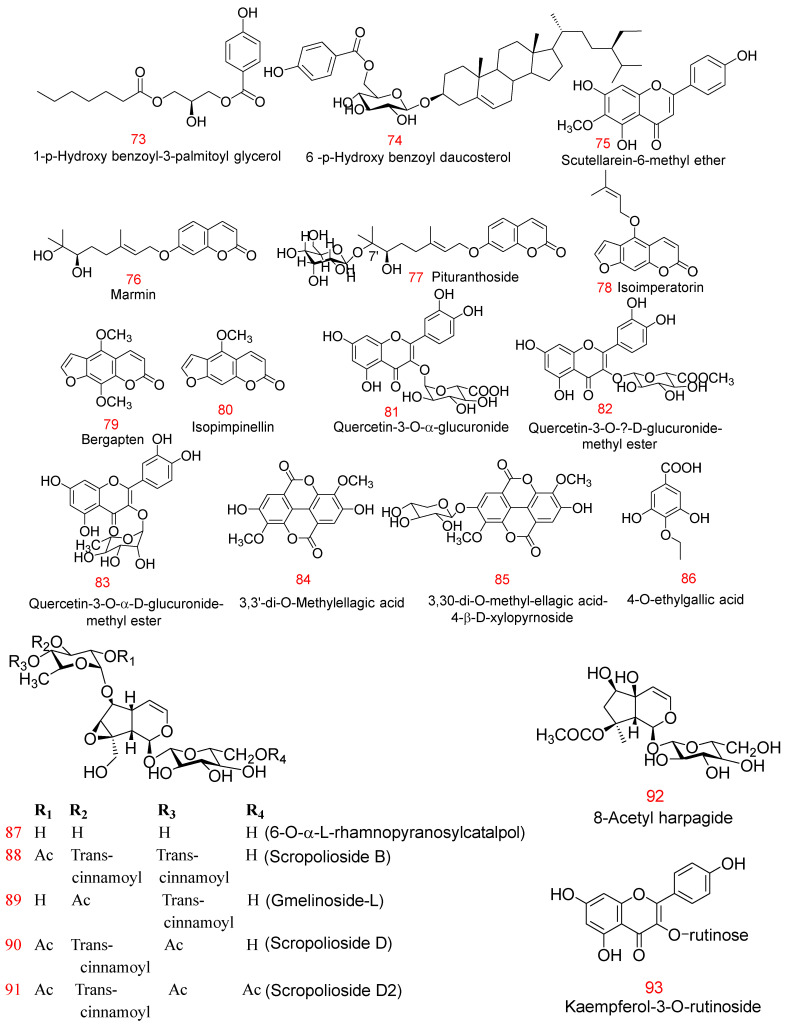

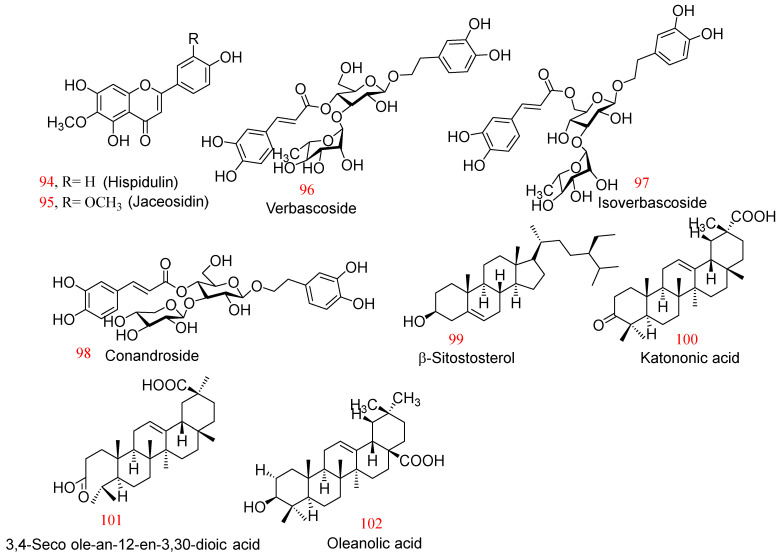
Structures of compounds **19–102** with different biological activities.

Over a million years, microbes have been present on this planet—developing, surviving, and adapting to environmental changes throughout the eras and causing many infectious diseases [108]. With the discovery of antibiotics 70 years ago, innumerable human lives have been saved [109]. Antibiotics can kill or inhibit the growth of bacteria through various mechanisms (e.g., by inhibiting the final crosslinking stage of cell wall synthesis and forming an irreversible covalent bond with enzymes, resulting in cell wall weakening and cell death) [110]. In addition, antibiotics can act through the inhibition of nucleic acid (DNA and RNA) synthesis, protein synthesis, or disruption of cell membrane function [110,111]. Through the overuse antibiotics, bacteria have acquired resistance via mutations, biofilm formation, target protection, or acquisition of inherent genes, such as β-lactams, fluoroquinolones, and aminoglycosides. Moreover, bacteria can convert antibiotics to their inactive forms via hydrolysis or via the addition of chemical groups using enzymes, leading to the dramatic emergence of multidrug-resistant pathogens and the rapid spread of new infections [111,112,113,114]. In the U.S. in August 2016, the first case was reported where a patient with an infection caused by *Klebsiella pneumoniae* was resistant to 26 antibiotics, including all aminoglycosides and polymyxins tested, and was intermediately resistant to tigecycline, resulting in the death of the patient [115]. The most pathogenic bacteria that threaten human health are *Enterococcus faecium*, *Staphylococcus aureus*, *K. pneumoniae*, *Acinetobacter baumannii*, *Pseudomonas aeruginosa*, and *Enterobacter* species termed the ‘ESKAPE pathogens’ [116]. These microorganisms are usually associated with an increased economic burden compared with those that are sensitive or have no potential to infect or colonise humans [117]. If there is no action today, there will be no treatment tomorrow, as the dramatic increase in drug resistance without a solution will result in one person dying every 3 s from antibiotic resistance by 2050 [118]. Consequently, pharmaceutical and scientific communities are searching for alternative sources, and natural antimicrobial plant-derived substances will be a powerful weapon against pathogens. Plants are a significant sources of secondary metabolites with antimicrobial properties, such as saponins, terpenoids, alkaloids, tannins, alkenyl phenols, flavonoids, glycoalkaloids, sesquiterpenes lactones, and phorbol esters, which help to inhibit and slow the development of resistance in addition to their other activities [119,120,121,122,123]. Unfortunately, only a few new antimicrobials have been reported out of an estimated 250,000 to 500,000 plant species on Earth [114]. Recently, it was reported that 48.7% of people use antibiotics in SA, making it the second most used drug in SA [8]. More seriously, most consumers purchase antibiotics without prescription. For example, in Riyadh city, three-quarters of the examined pharmacies have sold antibiotics without a prescription to consumers, which has greatly affected patients and caused in increase in resistant organisms [124]. However, in April 2018, the Ministry of Health in SA banned the purchase of antibiotics without a prescription [125]. Some plant extracts and isolated active compounds act to combat microbial resistance through different mechanisms as showed in Figure 5. For example, *Nigella sativa* ethanolic extract showed promising results against 99 strains of methicillin-resistant *S. aureus* at 4 mg/disc concentration, with MIC 0.2–0.5 mg/mL [121,126]. In addition, active compounds from *Psiadia punctulata* surface extract have antimicrobial activity, with the ability to reduce the biofilm formation of *Candida albicans* and *S. aureus* by 90% and 50%, respectively, at a concentration of 40 μg/mL [77]. Recently, Biharee et al. reported the importance of flavonoids as antimicrobial agents, as they impair bacterial efflux pumps, inhibit biofilm formation, disrupt the cell membrane, and prevent cell envelope formation [127]. For example, when the total content of flavonoids of 16 cultivars of dates that are grown in SA were analysed, their content varied from 27 to 199 mg CE/100 g dry weight (DW) [128]. This indicates the potentially potent antibacterial activity against imipenem-resistant *P. aeruginosa* of flavonoid glycosides isolated from *Phoenix dactylifera* L. (Tamar). All 12 tested strains were sensitive to the flavonoid glycosides at MIC and MBC values of 0.5–1 mg/mL. Scanning electron microscopy revealed that cell morphology was distorted after 60 min of flavonoid glycoside treatment, explained as a result of damage to the *P. aeruginosa* cell wall and the formation of pores. Moreover, flavonoid glycosides inhibited biofilm formation after 1 h of exposure. According to the authors, the possible mode of action is associated with the distribution of the phospholipid bilayers in the bacterial cell membrane [129]. In another study, the methanol extract of fruits, aerial parts, leaves, and resins of 17 plants were evaluated for their antimicrobial activity; *Chenopodium murale* L., *Commiphora myrrha*, *Euphorbia helioscopia* L., and *Solanum incanum* L. had MICs of 2.5 mg/mL against *Staphylococcus carnosus* and *Escherichia coli* (standard = 0.01 mg/mL) [130]. Aqueous, methyl alcohol, and methanol extracts of *S. persica* L. from different regions in SA were evaluated for their activity against various organisms, such as *S. aureus*, *Lactobacillus acidophilus*, *Streptococcus mutans*, *P. aeruginosa*, *Fusobacterium nucleatum*, *Lactobacillus casei*, *Staphylococcus epidermidis*, and *Streptococcus salivarius*. Their inhibition zones ranged from 9.2 to 29 mm compared with the positive control (15.4–34.0 mm). The potent activity was related to the active components of *S. persica*, including flavonoids, salvadorine, alkaloids, cyanogenic, salvadourea, glycosides, and tannins, which are known to possess strong antimicrobial activity [131,132,133]. Methanolic extracts from the leaves, stem, flower, and fruit of *Conocarpus erectus* L. and the leaves of *Aerva javanica* exhibited strong antibacterial activity against *Bacillus subtilis*, *S. aureus*, and the acid-fast bacterium *Mycobacterium phlei* [134,135,136]. Furthermore, the methanolic extracts of *Mentha* × *piperita* and *Mentha longifolia* populations grown in northern SA showed that *M.* × *piperita* contained the flavonoids naringin 328.8 mg/100 g DW, cynaroside 162.8 mg/100 g DW, and higher phenolic acid content than *M. longifolia*: rosmarinic acid 1547.6 mg/100 g DW, cryptochlorogenic acid 91.7 mg/100 g DW, and chlorogenic acid 69.4 mg/100 g DW. The two *Mentha* species had potent antioxidant, anti-cancer, and antimicrobial activities attributed to their polyphenols [137,138]. Carvacrol (the main compound in both the volatile oil and aqueous distillates of aerial parts of *Origanum vulgare*) inhibited the growth of *E. coli* at 200 µg/mL with an IC_50_ of 42 µg/mL compared with the control (20 µg /mL), causing cell membrane damage and cell death [139]. Ethanolic extract of various parts of *Syzygium aromaticum*, *Punica granatum*, *Thymus vulgaris*, *Cuminum cyminum*, and *Zingiber officinales* exhibited superior activity against bacterial strains that cause diseases of food poisoning [140]. Moreover, it was revealed that the essential oils of *Artemisia* species (*A. absinthium*, *A. sieberi*, and *A. scoparia)* contain four common compounds: limonene, camphor, terpinen-4-ol, and ethyl 2-methylbutyrate. *Artemisia* species exhibited good antimicrobial activity against *S. aureus* CP011526.1, *Bacillus licheniformis* KX785171.1, and *Micrococcus luteus* NCTC 2665 bacterial strains and *C. albicans* MF942350, *Candida parapsilosis* MF942354, and *Aspergillus parasiticus* CBS 100926 fungal strains, with MICs ranging from 0.1 µg/mL to 11.0 µg/mL [141]. Methanolic extracts of *Pimpinella anisum*, *Curcuma longa*, *Zingiber officinale*, and *Commiphora molmol* exhibited significantly higher antimicrobial activity than aqueous extracts when compared with kanamycin as a positive control [142] in addition to other active compounds with potent activity, as illustrated in Table 3. Although SA is home to numerous wild plant species with significant antibacterial activity, few studies have addressed the mechanisms and modes of their action. Therefore, there is a need for in-depth studies to validate the importance of these plant species as antimicrobial agents.

## 7. Clinical Trials of *Nigella sativa* (Black Cumin Seeds), a Plant Introduced into SA, with Other Plants Recommended for Clinical Trials

The Prophet Muhammad (PBUH) said that ‘In the black cumin, there is a cure for every disease except death’ [148]. In SA, *N. sativa* L. is commonly cultivated in the central and western regions [149]. In 2030, SA is expected to be the 17th and 12th among the top 50 countries/territories for diabetes and obesity prevalence, respectively [150]; about 60% of diabetes cases, especially type 2 diabetes, can be directly attributed to obesity [151]. *N. sativa* supplementation was evaluated for its potent activity in various studies on diabetic Saudis. When 92 patients with type 2 diabetes took different doses of *N. sativa* (1, 2, and 3 g) daily for 3 months, their blood glucose significantly decreased at a dose of 2 g/day [152]. Furthermore, healthy volunteers in another survey exhibited a decrease in blood glucose level after 2 weeks of taking 2 g/day of *N. sativa*, indicating that this is an optimal dose [153]. Therefore, a study evaluated the effect of 2 g/day of *N. sativa* orally for 1 year in 57 type 2 diabetic patients in addition to oral hypoglycaemic agents. In comparison with the control group (57 patients with the same regimen of placebo), there was a significant decrease in the total cholesterol (TC), blood pressure, and heart rate of the patients who took *N. sativa*. The decrease in TC, low-density lipoprotein (LDL-C), TC/high-density lipoprotein (HDL-C) and LDL-C/HDL-C ratios, diastolic blood pressure, systolic blood pressure, and mean arterial pressure compared with controls reflect the protective role of *N. sativa* against cardiovascular diseases associated with diabetics, without any reported side effects [154]. This cardiovascular activity confirmed the data of Bamosa and colleagues, where 30 diabetic patients received the same dose of *N. sativa* (2 g/day) for 1 year, showing a protective effect against cardiac diastolic by enhancing the systolic function of patients [155]. Moreover, glycaemic control improved as fasting blood glucose and HbA1c were reduced in all readings over the year, which may be related to enhanced insulin secretion by *N. sativa* [156]. Patients with diabetic nephropathy are at an increased risk of chronic kidney diseases (CKD). In a study, 32 patients with CKD stages 3 and 4 due to DN received 2.5 mL of *N. sativa* oil once orally every day for 12 weeks, and antidiabetic properties were reported. The patients’ glycaemic control and renal function improved due to a reduction in blood glucose, blood urea, serum creatinine, and 24 h total protein levels in the urine in addition to an increase in haemoglobin level, glomerular filtration rate, and total urinary volume in 24 h, with few side effects compared with patients who received only conservative drugs [157]. *N. sativa* was reported to have anti-asthmatic effects in a clinical study on 76 asthma patients; 24 patients received a placebo only (control group), and the rest were divided into two groups and received different doses of *N. sativa* (1 and 2 g/day) for 3 months. An increase in both forced expiratory flow (25–75%) and forced expiratory volume at 1 s was observed in the group treated with 2 g per day of *N. sativa* in addition to an improvement in peak expiratory flow and serum IFN-γ in both groups. Serum IgE and FeNO decreased in both groups compared with the control group, reflecting the ability of *N. sativa* to control asthma by reducing bronchitis [158].

**Figure 6:**
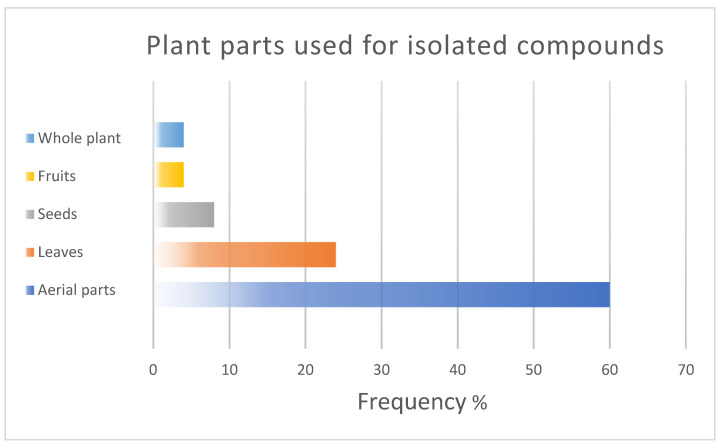
Plant parts that are commonly used for the isolation of bioactive compounds.

**Figure 7:**
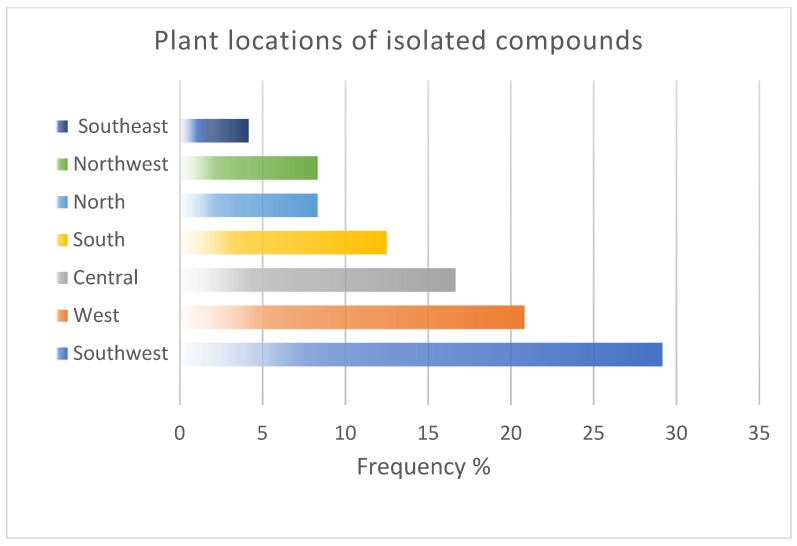
Common plant locations, as described in this review.

In SA, studies have focused on the effects of *N. sativa* in diabetic patients; however, other clinical studies on Saudi Arabian plants have reported potent diabetic effects in vivo, including *Ricinus communis* L. [159], *Caralluma sinaica* (Decne.) A. Berger [160], *Murraya koenigii* L., *Olea europaea* L. [161], *Rosmarinus officinalis* L. [162], *Hammada saticornica*, *Teucriurn Oliverianum*, and *Allium cepa* L. [163], in addition to the reported antidiabetic effects of various active compounds [164]. Furthermore, *N. sativa* has been reported to have various antimicrobial, anti-inflammatory, antitumour, and antioxidant clinical activities [165], while clinical studies in SA focus only on diabetes. Many other plants have been recommended in the Holy Qur’ân and Ahadith [50], including the date. Prophet Muhammad (PBUH) stated: ‘Whoever ate seven Ajwa dates in the morning ‘from the area of ،Aaliyah’ will not be harmed by poison or magic the rest of that day’ [166]. Even though SA is considered the third-largest producer of dates after Egypt and Iran [167], there are no clinical studies to confirm its activities compared with those of synthetic drugs. In addition, most of the studies focused on compounds isolated from the aerial parts mainly leaves of plants from the western and southwestern regions as in Figure 6 and Figure 7 which encourages the study of different plant parts throughout the kingdom.

## 8. Conclusions and Future Directions

SA features unique characteristics, including climate, location, geographical nature, and religion. These environmental and social conditions have resulted in SA becoming home to plant species with high biodiversity. The predominantly desert nature and continuous movement of nomads have enriched the traditional uses of plants and ethnopharmacology as part of the cultural heritage. About 102 isolated active compounds have been reported as leading compounds for the treatment of serious diseases, including cancer, inflammation, diabetes, and microbial infections. Most of these compounds are isolated from the aerial parts (mainly leaves) of plants from the western and southwestern regions, especially in the spring and winter. Their modes of action, mechanisms, and synthesis strategies require further investigation to obtain an in-depth understanding of these precious resources and their bioactive compounds. The clinical trials for *N. sativa* against diabetes are ongoing and show great promise. Other plant families, including Asteraceae and Apocynaceae, are being developed in clinical trials against cancer, paving the way for new potential therapeutic entities. Conservation and management of these valuable species is highly recommended and requires the cessation of anthropogenic activities that harm the distinctive and unique characteristics of SA that allow these species to flourish.

## Figures and Tables

**Table 1:** The biological activities of isolated compounds from plants in the central and western regions of SA.

S. No.	Plant/Family	Location/Time	Solvent/Part Used	Animal, Cell Line, Organism, or Method Used	Activity	Ref.
**23**	*Premna resinosa* (Hochst.) Schauer/Lamiaceae	Riyadh (central)/Jan. 2011 region	Methanol extract/aerial parts	Daoy, HepG2, and SK-MEL28/DPPH Scavenging activity (DPPH SCA) assay	Cytotoxic activity just against Daoy cell line with IC_50_ = 10.5 μg/mL PC = 7.3 μg/mL Antioxidant activity via inhibition of the discoloration of β-carotene by 94.4% at 1000 μg/mL PC = 93.1%	[71]
	*Commiphora opobalsamum* L./Burseraceae	Near Abha city (Southern)/2003	Various fractions/aerial parts	DCFH-DA method/HL-60 cells	Antioxidant activity with IC_50_ = 0.55 μg/mL PC = 0.39 μg/mL	[72]
**24**	*P. resinosa* (Hochst.) Schauer/Lamiaceae	Riyadh (central)/Jan. 2011	Methanol extract/aerial parts	HepG2, Daoy, and SK-MEL28/DPPH SCA assay	Cytotoxic activity just against Daoy cell line with IC_50_ = 21.1 μg/mL PC = 7.3 μg/mL Antioxidant activity via inhibition of the discoloration of β-carotene by 91.2% at 1000 μg/mL PC = 93.1%	[71]
**25**	*P. resinosa* (Hochst.) Schauer/Lamiaceae	Riyadh (central)/Jan. 2011	Methanol extract/aerial parts	HepG2, Daoy, and SK-MEL28/DPPH SCA assay	Cytotoxic activity just against Daoy cell line with IC_50_ = 7 μg/mL PC = 7.3 μg/mL Antioxidant activity via inhibition of the discoloration of β-carotene by 80.9% at 1000 μg/mL PC = 93.1%	[71]
**26**	*P. resinosa* (Hochst.) Schauer/Lamiaceae	Riyadh (central)/Jan. 2011	Methanol extract/aerial parts	Daoy, HepG2, and SK-MEL28/DPPH SCA assay	Cytotoxic activity just against Daoy cell line with IC_50_ = 19.6 μg/mL PC = 7.3 μg/mL Antioxidant activity via inhibition of the discoloration of β-carotene by 90.5% at 1000 μg/mL PC = 93.1%	[71]
**1**	*P. resinosa* (Hochst.) Schauer/Lamiaceae	Riyadh (central)/Jan. 2011	Methanol extract/aerial parts	Daoy, HepG2, and SK-MEL28/DPPH SCA assay	Cytotoxic activity just against SK-MEL28 cell line with IC_50_ = 18.8 μg/mL PC = 23.8 μg/mL Antioxidant activity via inhibition of the discoloration of β-carotene by 86.2% at 1000 μg/mL PC = 93.1%	[71]
**38**, **39**, **40**, **41**, **42**, **43**, **44**, **45**, **24**, and **46**	Sisymbrium irio L/Cruciferae	Najed Region (central)/Apr. 2008	Ethanol extract/aerial parts	Scavenging of the ABTS radical	Antioxidant activity ranged from 372 to 785 μM Trolox equivalent/g dry weight	[73]
**52**, **53**, **54**, **47**, **55**, **56**, **35**, **57**, and **58**	*Cordia sinensis*/Boraginaceae	Riyadh (Central)	Methanol extract/dried plant	Carrageen-induced paw oedema of rats	Anti-inflammation-inhibited oedema ranged from 38.4 to 62.4% PC = 57.6% Anti-glycation activity ranged from 68 to 88.4% PC = 86%	[74]
**68**	*Sarcocornia fruticose*/Chenopodiaceae	Al-Kharrar lagoon-Red Sea, Jeddah (west)/Apr. 2016	Methanol extract/leaves	HCV Protease inhibitory assay	Showed inhibition of HCV protease with IC_50_ = 8.9 μM PC = 1.5 μM	[75]
**69** and **70**	*S. fruticose*/Chenopodiaceae	Al-Kharrar lagoon-Red Sea, Jeddah (west)/Apr. 2016	Methanol extract/leaves	DPPH SCA assay	Antioxidant activity with IC_50_ = 3.8 and 4.3 μM of compounds 69 and 70, respectively PC = 1.5 μM	[75]
**71**	*P. punctulata*/Asteraceae	Al-Kharrar lagoon-Red Sea, Jeddah (west)/Apr. 2016	Methanol extract/root	Jurkat and HeLa cell lines/	The antiproliferative activity caused growth inhibition with IC_50_ = 12 and 18 µM of Jurkat and HeLa cells, respectively, due to inducing cell cycle arrest in G0/G1. Lowering the basal level of peroxides DHFDA-load in the cell.	[75]
**73**, **74**, **75**, **23**, and **60**	*Cassia italica*/Fabaceae	Gabal Al-Ateeq, Al Madinah Al Munawwarah (west)/Apr. 2017	Ethyl acetate fraction/aerial parts	DPPH assay	Potent antioxidant activity ranges from 19.7 to 95.8%, compared to butylated hydroxyanisole 93.8%	[76]
** 37 **	*Psiadia punctulata/*Asteraceae	Wadi Ghazal (west)/Jun. 2012	Dichloromethane surface extract/leaves	Biofilm inhibition against *C. Albicans* and *S. aureus*	Reduce the biofilm formation of both *S. aureus* and *C. Albicans* at 40 μg/mL concentration by 50% and 90%, respectively	[77]

PC: positive control.

**Table 2:** The biological activities of isolated compounds of plants grown in the southwestern regions of SA.

S. No.	Plant/Family	Location/Time	Solvent/Part Used	Animal, Cell Line, Organism, or Method Used	Activity	Ref.
**20**	*Anvillea garcinii* (Burm.f.) DC./Asteraceae	15 km southwest of Al-Kharj city/Feb. 2016	Ethanol extract/leaves	*E. fergusonii*, *C. albicans*, *E. xiangfangensis*, *S. aureus*, *B. licheniformis*, *C. parapsilosis*, and *P. aeruginosa*	MIC_50_ = 32.3 and 22.3 mg/mL against *C. albicans*, *and C. parapsilosis* PC = 54.7, 51.5 mg/mL, respectively MIC_50_ = 0.32, and 1.7 mg/mL against *S. aureus and E. fergusonii* PC = 0.523 μg /mL	[102]
**21**	*A. garcinii* (Burm.f.) DC./Asteraceae	15 km southwest of Al-Kharj city/Feb. 2016	Ethanol extract/leaves	*C. parapsilosis*, *S. aureus*, *B. licheniformis*, *C. albicans*, *E. fergusonii*, *E. xiangfangensis*, and *P. aeruginosa*	MIC_50_ = 41.6 and 34.9 mg/mL against *C. albicans*, *and C. parapsilosis* PC= 54.7, 51.5 mg/mL, respectively MIC_50_ = 1.4, and 3.5 mg/mL against *S. aureus and E. fergusonii* PC = 0.523 μg /mL	[102]
**27**	*Plectranthus barbatus* Andrews/Labiatae	Al-Namas area in Asir (southwest)	Ethanol extract/aerial parts	Hela, HepG2, and HT-29	IC_50_ ranged from 15.10 to 26.58 μg/mL PC = 6.31–28.19 μg/mL	[103]
				*S. aureus*, *S. mutans*, *E. coli*, and *S. typhi.*	Antibacterial activity against *S. aureus*, *S. mutans* with MIC= 31.25 μg/mL for both PC = 7.8 μg/mL	
				*Penicillium aurantiogriseu*, *Aspergillus niger*, *Candida albicans*, and *Cryptococcus neoformans*	Antifungal activity with MIC= 64.5 μg/mL against all except for *c. neoformans* 31.25 μg/mL PC = 1.75–7 μg/mL	
				DPPH radical-scavenging activity	Total antioxidant activity at 1000 μg/mL = 69.2% PC = 87.1 μg/mL	
**28**	*P. barbatus* Andrews/Labiatae	Al-Namas area in Asir (southwest)	Ethanol extract/aerial parts	Hela, HepG2, and HT-29	IC_50_ ranged from 41.23 to 69.55 μg/mL PC = 6.31–28.19 μg/mL	[103]
				*E. coli*, *S. aureus*, *S. mutans*, and *S.typhi.*	Antibacterial activity against *S. aureus*, *S. mutans* with MIC= 31.25 μg/mL for both PC = 7.8 μg/mL	
				*A. niger*, *P. aurantiogriseum*, *C. albicans* and *C. neoformans*	Antifungal activity with MIC= 64.5 μg/mL against all except for *c. neoformans* 31.25 μg/mL PC = 1.75–7 μg/mL	
**29**	*P. barbatus* Andrews/Labiatae	Al-Namas area in Asir (southwest)	Ethanol extract/aerial parts	Hela, HepG2, and HT-29	IC_50_ ranged from 40.72 to 72.75 μg/mL PC = 6.31–28.19 μg/mL	[103]
				*E. coli*, *S. aureus*, *S. mutans*, and *S. typhi.*	Antibacterial activity with MIC= 15.6 μg/mL against all except for *E-coil* 31.25 μg/mL PC = 3.9–7.8 μg/mL	
				*P. aurantiogriseum*, *A. niger*, *C. albicans* and *C. neoformans*	Antifungal activity with MIC= 15.6–64.5 μg/mL PC = 1.75–7 μg/mL	
				DPPH SCA assay	Total antioxidant activity at 1000 μg/mL= 25.9% PC = 87.1 μg/mL	
**30**	*P. barbatus* Andrews/Labiatae	Al-Namas area in Asir (southwest)	Ethanol extract/aerial parts	*S. aureus*, *S. mutans*, *E. coli*, and *S. typhi.*	Antibacterial activity with MIC= ranged from 15.6 to 31.25 μg/mL PC = 3.9–7.8 μg/mL	[103]
				*A. niger*, *P. aurantiogriseum*, *C. albicans and C. neoformans*	Antifungal activity with MIC= 31.25–64.5 μg/mL PC = 1.75–7 μg/mL	
				DPPH SCA assay	Total antioxidant activity at 1000 μg/mL = 64.8% PC = 87.1 μg/mL	
**34**	*Cadaba farinosa* Forssk./Capparidaceae	Al-Baha (southwest)/Mar.2010	Methanol extract/leaves	DPPH SCA assay	Antioxidant activity by 87.1% PC = 100%	[104]
**35**	*C. farinosa* Forssk./Capparidaceae	Al-Baha (southwest)/Mar.2010	Methanol extract/leaves	DPPH SCA assay	Antioxidant activity by 71.8% PC = 100%	[104]
**36**	*C. farinosa* Forssk./Capparidaceae	Al-Baha (southwest)/Mar.2010	Methanol extract/leaves	DPPH SCA assay	Antioxidant activity by 49.1% PC = 100%	[104]
**62**, **63**, **64**, **65**, **66**, and **67**	*Tagetes minuta* L./Asteraceae	Al-Baha (southwest)/Mar.2015	Methanol extract/aerial parts	DPPH SCA assay/ IL-6 and TNF-α level measurement	The antioxidant activity showed an 87.13–93.62% scaving activity at 100 μM. Reduction in NFκB p65, IL-6, and TNF-α levels in PBMCS	[105]
**72**	*Dobera glabra* (Forssk)/Salvadoraceae	Shoqaiq (southwest)/Feb. 2013	Ethanol extract/aerial parts	PC-3, HepG-2, and HCT-116 cancer cell lines	High cytotoxicity against all cell lines with IC_50_ = 8 μg/mL	[106]
**23**, **81**, **82**, **83**, **84**, **85**, and **86**	*Euphorbia schimperiana/*Euphorbiaceae	Al-Sawda in Asir region (southwest)/Feb. 2018	Ethyl acetate and *n*-butanol/aerial parts	HCT116, MCF7, HePG2, and PC3 cancer cell lines *Listeria monocytogenes* Scott A, *S. aureus* (ATCC 6538), *B. cereus*, *E. faecalis*, *S. enterica*, *P. aeruginosa*, *K. pneumonia*, and *E. coli* (ATCC 8739)	Compound 84 showed potent cytotoxic activity against PC3 with IC_50_ = 5.5 μg/mL Compounds 83,85 and 86 exhibited antibacterial activity against all tested strains	[107]

PC: positive control.

**Table 3:** The biological activities of isolated compounds from plants in various regions of SA.

S. No.	Plant/Family	Location/Time	Solvent/Part Used	Animal, Cell Line, Organism, or Method Used	Activity	Ref.
**19**	*Origanum vulgare L./*Lamiaceae.	Al-Kharj (southeast)/Mar. 2013	Oils/aerial parts	*E. coli*, *M. luteus*, *P. aeruginosa*, and *S. aureus*	Carvacrol completely inhibited the growth of *E. coli* at 200 μg. mL^−1^ with IC_50_ = 42 μg/mL PC = 20 μg/mL	[139]
**22**	*Myrtus* *communis* Linn./Myrtaceae	District Sawat (northwest)/Jul. to Aug. 2016	Acetone extract/leaves	*E. coli* and *S. aureus*	Inhibition zone (IZ) of the compound = **36**, and 37.5 mm compared to acetone extract 16, 15.5 mm against *E. coli* and *S. aureus*, respectively, at 50 mg/mL	[143]
**31**	*Commiphora opobalsamum* L./Burseraceae	Near Abha city (southern)/2003	Various fractions/aerial parts	Chloroquin-sensitive (D6) and chloroquin-resistant (W2) (strains of P. falciparum).	Antimalarial activity with IC_50_ = 3.5 and 3.2 μg/mL against D6 and W2 respectively PC = 9, and 10.5 ng/mL	[72]
**32**	*C. opobalsamum* L./Burseraceae	Near Abha city (southern)/2003	Various fractions/aerial parts	DCFH-DA method/HL-60 cells	Antioxidant activity with IC_50_ = 5.00 μg/mL PC = 0.39 μg/mL	[72]
**33**	*C. opobalsamum* L./Burseraceae	Near Abha city (southern)/2003	Various fractions/aerial parts	DCFH-DA method/HL-60 cells	Antioxidant activity with IC_50_ = 0.70 μg/mL PC = 0.39 μg/mL	[72]
**47**	*Gastrocotyle hispida*/Boraginaceae	Hail (northwest)/Mar. 2015	Methanol extract/aerial parts	DPPH SCA assay	Antioxidant activity with IC_50_ = 10.2 μg/mL, PC = 8.1 μg/mL	[144]
				MDA-MB 231, HEK-293, CF-7, and HepG2	Anticancer activity against MCF-7, and HEK-293 with IC_50_ = 4.2 and 5 μg/mL, PC = (3.2–2.4) μg/mL	
**48**	*G. hispida*/Boraginaceae	Hail (northwest)/Mar. 2015	Methanol extract/aerial parts	DPPH SCA assay	Antioxidant activity with IC_50_ = 52 μg/mL, PC = 8.1 μg/mL	[144]
				MDA-MB 231, HEK-293, CF-7, and HepG2	Anticancer activity against HEK-293, and MCF-7 with IC_50_ = 2.3 and 6.2 μg/mL, PC = (3.2–2.4) μg/mL	
**49**, **50**, and **51**	*G. hispida*/Boraginaceae	Hail (northwest)/Mar. 2015	Methanol extract/aerial parts	MDA-MB 231, HEK-293, CF-7, and HepG2	Anticancer activity with IC_50_ ranges from 3.7–71 μg/mL PC = 2.1–3.2 μg/mL	[144]
**59**, **40**, **60**, and **61**	*Chrozophora tinctoria*/Euphorbiaceae	Al-Hadda road/Apr. 2012	Methanol extract/aerial parts	Measurement of TNF-α, PGE_2_, IL-1b, IL-6, and levels	Anti-inflammation activity by decreasing the level of Il-1b, Il6, PGE_2_, and TNF-α in the supernatant media of human peripheral blood mononuclear cells (PBMCS)	[92]
**76**, **77**, **78**, **79**, and **80**	*Deverra tortuosa* (Desf.)/Apiaceae	Wadi Abalkour (north)/Sept. 2017	*n*-Hexane and ethyl acetate/seeds	α-Glucosidase inhibitory test. *E. Coli* (ATCC 25922), *K. pneumonia* (ATCC 700603), *Proteus mirabilis* (ATCC14153), *S. typhimurium* (ATCC 14028), *P. aeruginosa* (ATCC 254992), *S. aureus* (ATCC 254996) and *E.* *Faecalis* (ATCC 29212)	Compounds **76**, **77** and **78** exhibited high inhibition of α-glucosidse with IC_50_ = 0.05, 0.17, and 0.04 μg/μL, respectively, compared to acarbose (IC_50_ = 0.25 μg/μL) as a positive control Compound **77** showed the highest IZ values against the seven tested bacterial strains with IZ = 21 and 22 mm against *S. typhimurium* and *E. faecalis*, respectively, may be related to the presence of β-glucopyranose at the C-7′ position	[145]
**87**, **88**, **89**, **90**, **91**, **92**, **23**, and **93**	*Scrophularia syriaca* Benth./Scrophulariaceae	Aljouf region (north)/May 2016	Ethanol extract/aerial parts	*Trypanosoma brucei brucei* (s427-WT) *Trypanosoma brucei brucei* (TbAT1-B48) *Leishmania major* *Leishmania mexicana*	Compound **88** showed high antiprotozoal activity with EC_50_ ranging from 1.10 ± 0.18 μM to 8.34 ± 1.70 μM, while compounds **90**, **23**, and **93** exhibited mild-to-moderate activities, with EC_50_ ranging from 5.34 ± 0.52 to >100 μM compared to pentamidine	[146]
**94**, **95**, **96**, **97**, **98**, **99**, **100**, **101**, and **102**	*Nuxia oppositifolia* (Hochst)/Buddlejaceae)	Wadi Lajab (south)/Mar. 2015	Alcoholic extract/aerial parts	Radical scavenging activity Adulticidal activity	Compounds from **94** to **98** showed the ability to reduce the DPPH and (ABTS^+^) radicals with IC_50_ = ranging from 27.3 to 68.1 μg/mL Compounds **99, 100, 101**, and **102** exhibited adulticidal activity with LD_50_ values of 2–2.3 μg/mosquito	[147]

PC: positive control; CID: compound identifier; Puddumetin (PubChem CID: 5281617); kumatakenin (PubChem CID: 5318869); isorhamnetol (PubChem CID: 5281654); eupatorin (PubChem CID: 97214); Myricetin 3,3′,4′-Trimethylether (PubChem CID: 44259719); Cucurbitacine I glucoside (PubChem CID: 6441519); Cucurbitacine I glucoside (PubChem CID: 6441519); Carvacrol (PubChem CID: 10364 ); Quercetin ( PubChem CID: 5280343); Kaempferol (PubChem CID: 5280863); 3-Methoxy quercetin (PubChem CID: 318040061); Ferruginol (PubChem CID: 442027); Forskolin:(PubChem CID: 47936); Sugiol:(PubChem CID: 94162); Syringic acid: (PubChem CID: 10742); oleanolic acid: (PubChem CID: 10494); Friedelin:(PubChem CID: 91472); Mearnsetin: (PubChem CID: 10359384); 12- Aminododecanoic:(PubChem CID: 69661); Quercetin-3-O-β-D-lucopyranoside:(PubChem CID: 341713383); 5,7,3,4-Tetramethoxyflavone:(PubChem CID: 631170); Apigenin: (PubChem CID: 5280443); Apigenin-7-galactoside: (PubChem CID: 44257799); Apigenin-7-O-β-D-lucoside: (PubChem CID: 5280704 ); Luteolin-7-O-glucoside: (PubChem CID: 5280637); Rosmarinic acid:(PubChem CID: 5281792); β-Sitosterol:(PubChem CID: 160693018); Protocatechuic acid: (PubChem CID: 72); Trans-caffeic acid: (PubChem CID: 689043); Methyl rosmarinate: (PubChem CID: 6479915); Amentoflavone: (PubChem CID: 5281600); Rutin (PubChem CID: 5280805); p-Hydroxybenzoic acid: (PubChem CID: 135); Protocatechuic acid methyl ester: (PubChem CID: 287064); Gallic acid:(PubChem CID: 370); 7α-Hydroxystigmasterol: (PubChem CID: 101704480); Scutellarein-6-methyl ether: (PubChem CID: 5281628); Marmin: (PubChem CID: 6450230 ); Pituranthoside: (PubChem CID: 101682294); Isoimperatorin: (PubChem CID: 68081); Bergapten: (PubChem CID: 2355); Isopimpinellin: (PubChem CID: 68079); Quercetin-3-O-α-glucuronide: (PubChem CID: 5274585); Quercetin-3-O-α-L-rhamnoside: (PubChem CID: 5280459); 3,3′-di-O-Methylellagic acid: (PubChem CID: 5488919); Scropolioside B: (PubChem CID: 45360375); Scropolioside D:(PubChem CID: 6918700); Scropolioside D2: (PubChem CID: 6450157); 8-Acetyl harpagide: (PubChem CID: 9978650); Kaempferol-3-O-rutinoside: (PubChem CID: 5318767); Hispidulin: (PubChem CID: 5281628); Jaceosidin: (PubChem CID: 5379096); Verbascoside: (PubChem CID: 5281800); Isoverbascoside: (PubChem CID: 6476333); Conandroside: (PubChem CID: 11192794); β-Sitostosterol: (PubChem CID: 222284); Katononic acid: (PubChem CID: 9981416); Oleanolic acid: (PubChem CID: 10494).

## Data Availability

Not applicable.
